# Downregulation of the small GTPase SAR1A: a key event underlying alcohol-induced Golgi fragmentation in hepatocytes

**DOI:** 10.1038/srep17127

**Published:** 2015-11-26

**Authors:** Armen Petrosyan, Pi-Wan Cheng, Dahn L. Clemens, Carol A. Casey

**Affiliations:** 1 Department of Biochemistry and Molecular Biology, College of Medicine, Omaha, NE, USA; 2 Department of Internal Medicine, University of Nebraska Medical Center, Omaha, NE, USA; 3 Nebraska Western Iowa Health Care System, VA Service, Department of Research Service, Omaha, NE, USA

## Abstract

The hepatic asialoglycoprotein receptor (ASGP-R) is posttranslationally modified in the Golgi *en route* to the plasma membrane, where it mediates clearance of desialylated serum glycoproteins. It is known that content of plasma membrane-associated ASGP-R is decreased after ethanol exposure, although the mechanisms remain elusive. Previously, we found that formation of compact Golgi requires dimerization of the largest Golgi matrix protein giantin. We hypothesize that ethanol-impaired giantin function may be related to altered trafficking of ASGP-R. Here we report that in HepG2 cells expressing alcohol dehydrogenase and hepatocytes of ethanol-fed rats, ethanol metabolism results in Golgi disorganization. This process is initiated by dysfunction of SAR1A GTPase followed by altered COPII vesicle formation and impaired Golgi delivery of the protein disulfide isomerase A3 (PDIA3), an enzyme that catalyzes giantin dimerization. Additionally, we show that *SAR1A* gene silencing in hepatocytes mimics the effect of ethanol: dedimerization of giantin, arresting PDIA3 in the endoplasmic reticulum (ER) and large-scale alterations in Golgi architecture. Ethanol-induced Golgi fission has no effect on ER-to-Golgi transportation of ASGP-R, however, it results in its deposition in *cis-medial*-, but not *trans*-Golgi. Thus, alcohol-induced deficiency in COPII vesicle formation predetermines Golgi fragmentation which, in turn, compromises the Golgi-to-plasma membrane transportation of ASGP-R.

The Golgi is the ‘heart’ of the intracellular transportation system where proteins synthesized in the rough endoplasmic reticulum (ER) are processed and sorted before being transported to other cellular organelles or secreted. Golgi contains stacks of flattened ribbon-like structure with the orchestration of matrix (golgins) and resident proteins. The residential enzymes, glycosyltransferases and glycosidases, are responsible for processing of proteins, and golgins serve as the building blocks of the Golgi architecture and docking sites for the ER-derived vesicles^1^,^2^,^3^,^4^,^5^. Giantin is the largest (376 kDa) golgin in mammals and essential for cross-bridging cisternae during Golgi biogenesis^6^. It consists of a short C-terminal domain located in the Golgi lumen, where a disulfide bond connects two monomers to form an active homodimer, which is followed by a one-pass transmembrane domain and then a large (≥350 kDa) N-terminal region^6^,^7^. Recently, we have shown that in advanced prostate cancer cells, giantin is present primarily as a monomer due to downregulation of protein disulfide isomerase A3 (PDIA3), also known as ERp57, the enzyme that catalyzes giantin dimerization^8^. The existence of giantin in monomeric form significantly affects the nucleation of the Golgi cisternal membrane stacks and alters O-glycosylation, thereby reducing the susceptibility of these cells to Galectin-1-induced apoptosis^8^. We are still far from a complete understanding of the mechanisms of cancer-specific Golgi fragmentation, but the Golgi remodeling and disorganization under the stress^9^,^10^,^11^ and treatment with many pharmacological drugs^12^,^13^,^14^ are already well-studied. In addition, the recent observations shed light on the phenomenon of Golgi disassembly in neurons induced by ethanol administration^15^,^16^. In the last decades, increasing attention has been given to alcohol’s effect on hepatocytes, and it has been uncovered that chronic and acute ethanol exposure result in impairment of intracellular transportation^17^. For instance, alcohol treatment can inhibit Golgi-to-plasma membrane trafficking, which results in intracellular accumulation of newly synthesized proteins and causes hypertrophy and ballooning of hepatocytes^18^. Ethanol treatment also compromises ER-to-Golgi transport^19^,^20^ and reduces the number of motile vesicles^21^. While alcohol-induced Golgi disorganization in hepatocytes has been reported^22^,^23^,^24^,^25^, the underlying mechanisms are still poorly understood. Moreover, the link between Golgi disassembly and the altered protein transport to and through the Golgi remains enigmatic.

The asialoglycoprotein receptor (ASGP-R), an endocytotic cell surface receptor expressed by hepatocytes, is a well-studied protein. The function of hepatic ASGP-R is to remove potentially hazardous asialoglycoproteins containing terminal galactose or N-acetylgalactosamine from the circulation^26^. Additionally, the ASGP-R is also able to bind oligosaccharides terminated with sialic acid^27^. A deficiency of ASGP-R is correlated to the alteration of hepatic function, and the impaired expression of ASGP-R has been described in cancer, viral hepatitis, and cirrhosis^28^,^29^. Chronic ethanol administration results in reduced levels of ASGP-R at the plasma membrane^30^,^31^, however, neither the mechanism of its impaired trafficking nor intracellular redistribution has been analyzed. We hypothesize that key to this impaired plasma membrane transport is ethanol-altered Golgi function. Several independent studies have shown that Golgi integrity depends on the constitutive cycling of Golgi components through the ER^2^, and alteration of coat protein complex II (COPII) vesicle formation results in crucial disorganization of Golgi architecture^32^,^33^,^34^. We are interested in whether a connection exists between ethanol administration and alteration in formation of COPII, and whether these changes may cause fragmentation of the Golgi.

The human ASGP-R is composed of two polypeptides, designated H1 and H2. Both H1 and H2 have a half-life of approximately 12 h, and in hepatocytes H1 is 5–6 times more abundant than H2^35^. ASGP-R is synthesized in the rough ER, transported to the Golgi, and finally targeted to the sinusoidal plasma membrane. Following ligand binding, ASGP-R is delivered to the compartment of uncoupling receptor and ligand (CURL), where ASGP-R-ligand complexes dissociate^36^. The ligand is then transferred to the lysosome for degradation, while unoccupied ASGP-Rs may either be degraded or delivered back to the cell surface^37^. In rat liver hepatocytes, 20% of the intracellular ASGP-R was found in the rough ER, 30% in the Golgi, and 50% in the smooth ER and CURL. The intra-Golgi pool of ASGP-R, including *trans*-Golgi network, in HepG2 cells is approximately 20% of total^36^. In recent years, the study of ASGP-R has experienced a renaissance because of the capacity of this receptor to import large molecules across the cellular plasma membrane, thus implying ASGP-R-targeted anticancer drug delivery^38^,^39^. Therefore, understanding the biology of ASGP-R, especially the mechanism of its trafficking to the plasma membrane is critically important for improving this type of drug delivery strategy.

In this study, we have shown that giantin dedimerization is involved in ethanol-induced Golgi fragmentation. Also, we have defined the mechanism by which COPII vesicles function as a Golgi delivery system for PDIA3 and provided evidence that the effects of ethanol on Golgi morphology is initiated by dysfunction of the small GTPase, SAR1A. These findings support our hypothesis that alcohol-altered deficiencies in COPII vesicles results in Golgi fragmentation which, in turn, alters the Golgi-to-plasma membrane trafficking of ASGP-R.

## Results

### Hepatic ADH-generated metabolite(s) of ethanol is a major contributor of Golgi fragmentation

**Figure 1:**
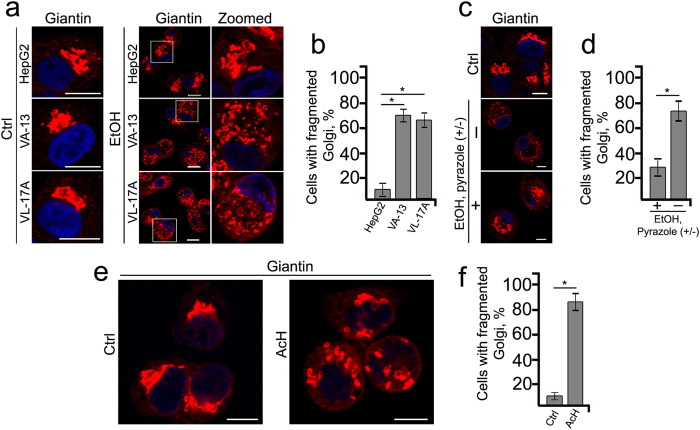
Hepatic ADH-generated metabolite of ethanol is a major contributor of Golgi fragmentation. (**a**) Immunostaining of giantin in HepG2 cells and recombinant HepG2 cells expressing ADH (VA-13) or ADH and CYP2E1 (VL-17A) after ethanol treatment (35 mM) for 72 h. (**b**) Quantification of fragmented Golgi in cells treated as described in (**a**); n = 30 cells for each series of experiments. (**c**) Immunostaining of giantin in VA-13 cells treated with ethanol in the presence or the absence of 5 mM pyrazole. (**d**) Quantification of cells with fragmented Golgi presented in (**c**); n = 40 cells for each series of experiments. (**e**) Immunostaining of giantin in VA-13 cells treated with 100 μM acetaldehyde (AcH) for 72 h. The control cells were treated with corresponding amount of PBS. (**f**) Quantification of cells with fragmented Golgi presented in (**e**); n = 30 cells. Nuclei were counterstained with DAPI (blue); bars, 10 μm. Results are expressed as a mean ± SD; *p < 0.001.

Hepatic alcohol dehydrogenase (ADH)-catalyzed oxidation of ethanol is the major pathway of ethanol metabolism in the body^40^. Hepatic microsomal cytochrome P450 (CYP2E1), which is induced by alcohol also can oxidize ethanol. To examine the metabolic basis of ethanol-induced cytotoxicity, we analyzed the Golgi morphology in HepG2 cells, which express very low levels of ADH, and two recombinant HepG2 cell lines: VA-13 cells that efficiently express ADH^41^ and VL-17A cells that express both ADH and CYP2E1^42^. After treatment with 35 mM ethanol for 72 h, the Golgi as monitored by giantin staining was distributed throughout the cell. The percentage of cells with fragmented Golgi in VA-13 and VL-17A cells was similar but significantly higher than that in the parental HepG2 cells (Fig. 1a,b). Because of the similar findings VA-13 cells were used for subsequent studies. Also, the effect of ethanol on Golgi morphology was confirmed by its co-staining with other Golgi markers, GRASP65 and GM130 (Supplementary Fig. S1). Next, when the ethanol treatment was carried out in the presence of 5 mM pyrazole, an ADH inhibitor^43^, the degree of Golgi fragmentation was significantly reduced (Fig. 1c,d). Furthermore, in response to treatment with 100 μM acetaldehyde for 72 h^44^, Golgi exhibits extensive disorganization (Fig. 1e,f). The results indicate that ethanol metabolites are the primary contributors to ethanol-induced Golgi fragmentation.

### Disassembly of the Golgi in SAR1A-depleted cells mimics the ethanol-induced phenotype

Formation of COPII vesicles is initiated by the small, highly conserved GTPase, SAR1, which, in turn, is a client protein for the ER resident guanine nucleotide exchange factor, Sec12. SAR1-GDP is converted to an active GTP-bound form at the ER membrane, followed by recruitment of Sec23-Sec24 and Sec13-Sec31 complexes^45^,^46^,^47^. Co-knockdown of both SAR1 isoforms, SAR1A and SAR1B, has been shown to disrupt the normal recruitment of the COPII vesicles, disseminate the ER-Golgi intermediate compartment (ERGIC) and fragment the Golgi^34^. Furthermore, *trans*-Golgi network exhibits as elongated structure without connection with Golgi stacks. In analogy to that, expression of GDP-restricted mutant of SAR1AT39N induces dispersion of Golgi^32^,^33^.

**Figure 2:**
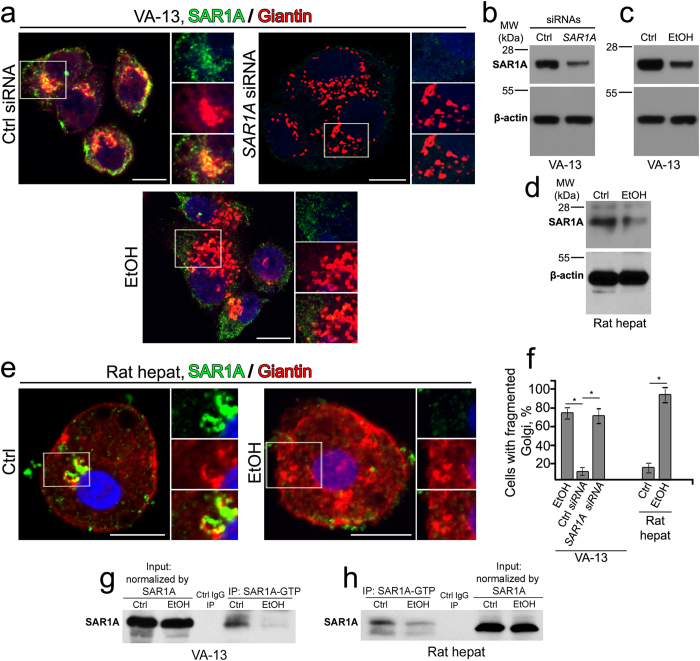
Ethanol treatment suppresses activation of SAR1A. (**a**) Immunostaining of SAR1A (green) and giantin (red) in VA-13 cells treated with 35 mM ethanol for 72 h, control siRNA or *SAR1A* siRNAs for 72 h. (**b**,**c**) SAR1A Western blot of the lysates of VA-13 cells treated with *SAR1A* siRNAs (**b**) or 35 mM ethanol for 72 h (**c**); β-actin was a loading control. (**d**) SAR1A Western blot of the lysates of hepatocytes isolated from ethanol-fed rats; β-actin was a loading control. (**e**) Immunostaining of SAR1A (green) and giantin (red) in hepatocytes isolated from ethanol-fed rats. The right panels show green, red and merged channels corresponding to the Golgi region (boxes). Nuclei were counterstained with DAPI (blue). All confocal images were acquired with same imaging parameters; bars, 10 μm. (**f**) Quantitation of fragmented Golgi in cells presented in (**a**) and (**e**); n = 35 cells for each series of experiments. The results shown are representative of three independent experiments and expressed as mean ± SD; *p < 0.001. (**g**,**h**) SAR1A Western blot of the complexes pulled down from the lysate of VA-13 cells (**g**) and hepatocytes (**h**) with anti-SAR1A-GTP Ab as described in Methods. The input protein was normalized by total SAR1A.

We sought to determine whether disruption of COPII function through depletion of SAR1A would result in Golgi disorganization similar to that we observed in ethanol-treated cells. In control cells, SAR1A-positive elements were detected as punctate sites, distributed throughout the cytoplasm, reflecting their predicted localization at the ER. Surprisingly, a substantial fraction of SAR1A was also present in the Golgi region as demonstrated by its colocalization with giantin (Fig. 2a). The Golgi in cells lacking SAR1A after *SAR1A* siRNAs treatment appeared scattered and dispersed in the cytoplasm, the phenotype, which resembles that observed in ethanol-treated cells (Fig. 2a,b,f). Importantly, both fluorescence microscopy and Western blotting showed that ethanol treatment led to decreased SAR1A (Fig. 2a,c), indicating that ethanol-induced Golgi disorganization might be initiated by downregulation of this protein. Interestingly, we detected that the decrease of SAR1A was apparent after 48 h treatment with ethanol, however at this time Golgi morphology remained intact, indicating that SAR1A downregulation precedes Golgi fragmentation (Supplementary Fig. S2a–c).

Similar to VA-13 cells, hepatocytes from ethanol-fed rats exhibited disorganized Golgi accompanied by a decrease of Golgi-specific SAR1A immunofluorescence and reduction of total SAR1A (Fig. 2d–f). Finally, we found that SAR1A activation was altered after ethanol administration in both VA-13 cells and rat hepatocytes, as determined by Western blot analysis, which showed a decrease of active GTP bound form of SAR1A (Fig. 2g,h).

Because cells experiencing SAR1 deficiency exhibit fragmented Golgi^34^, it is possible that SAR1A downregulation is the hallmark of Golgi disorganization. To test this, we treated VA-13 cells with the widely-used fungal metabolite Brefeldin A (BFA). Predictably, BFA disrupts structure of the Golgi^14^, however the expression of SAR1A was indistinguishable from that in untreated control (Supplementary Fig. S3a-c). These data suggest that SAR1-mediated mechanism cannot be simply ascribed to every type of Golgi fragmentation.

**Figure 3:**
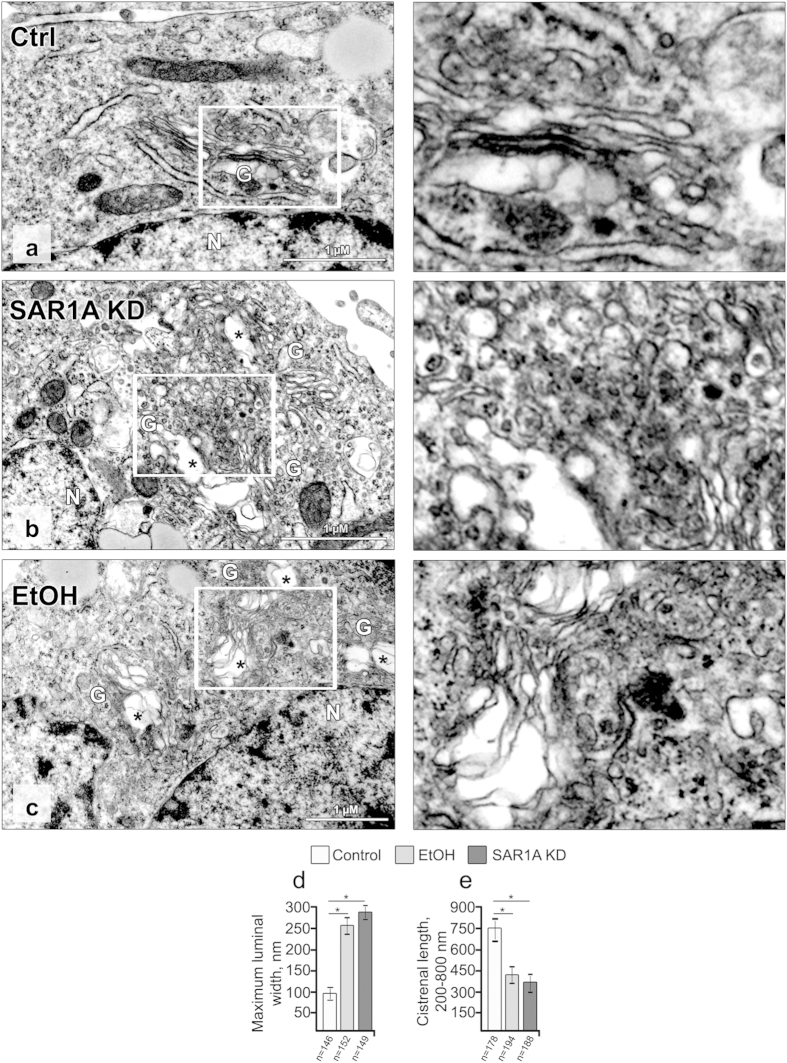
Depletion of SAR1A or treatment with ethanol results in significant fission of the Golgi. (**a**–**c**) Representative EM micrographs of VA-13 cells treated with control siRNA (**a**), *SAR1A* siRNAs (**b**) and 35 mM ethanol for 72 h (**c**). The Golgi fragments are presented as swollen membranes (asterisks) frequently observed adjacent to stacked, but shortened cisternae. White boxes indicate areas enlarged and shown at the right side. (**d**,**e**) Quantitation summarizing maximum luminal width of Golgi cisternal elements (**d**), and the average length of Golgi cisternae (from 200 to 800 nm). Calculations are performed by ImageJ software and results are expressed as a mean ± SD. Numbers (n) in (**d**) and (**e**) indicate the number of Golgi cisternae for which luminal width or length were measured; *p < 0.001. G, Golgi; N, nucleus.

To better understand the similarity of Golgi disorganization caused by SAR1A knockdown and ethanol, we examined Golgi morphology by electron microscopy. Contrary to the typical compact and stacked Golgi cisternae in control VA-13 cells, the Golgi in SAR1A-depleted and ethanol-treated cells were substantially disorganized (Fig. 3a–c). The main body of the Golgi was converted to several mini-Golgi structures exhibiting swollen and distended cisternae. We quantified the maximum luminal width of the Golgi cisternae and found that in ethanol-treated or SAR1A-depleted cells this parameter was significantly enhanced when compared with control cells (260 ± 15 nm in ethanol-treated and 280 ± 14 nm in SAR1A-depleted cells, vs. 98 ± 12 nm in control, Fig. 3d). Some Golgi cisternae were present as dilated vacuoles with round profiles (Fig. 3b,c, asterisks). Further, while stacking was still preserved in these Golgi structures, their cisternal length was greatly reduced. The average length of cisternae (ranging from 200 to 800 nm) was 750 ± 45 nm in control compared with 380 ± 50 and 350 ± 70 nm in ethanol-treated and SAR1A-depleted cells, respectively (Fig. 3e). The key question, then, is the mechanism that underlies the similarity of SAR1A-depleted and ethanol-induced Golgi disorganization.

### Ethanol treatment prevents giantin dimerization by blocking Golgi targeting of protein disulfide isomerase A3 (PDIA3)

**Figure 4:**
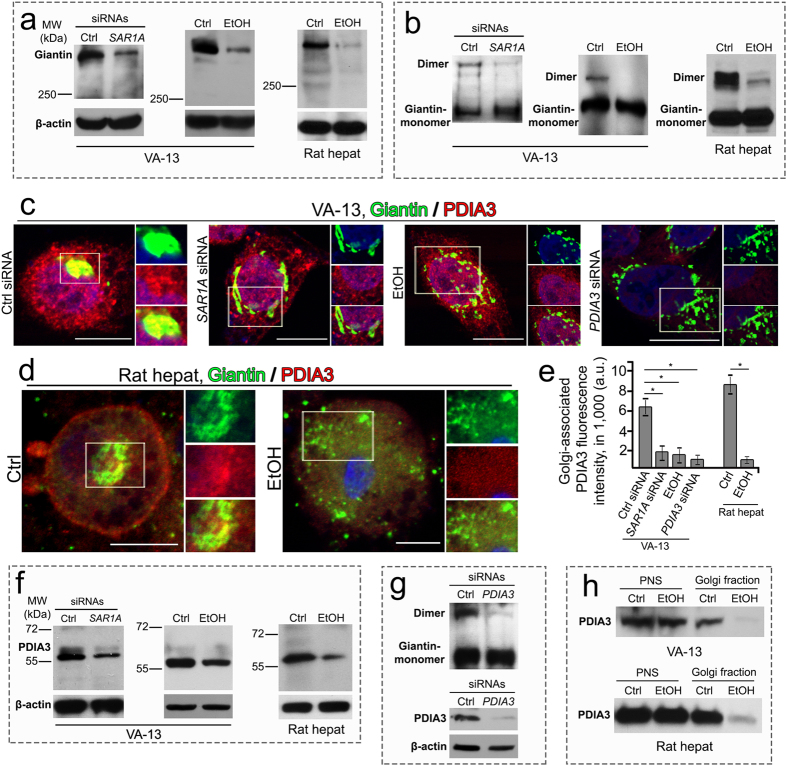
The effect of ethanol treatment or silencing *SAR1A* on expression and localization of protein disulfide isomerase A3 (PDIA3). (**a**,**b**) Giantin Western blot of the lysates of VA-13 cells treated with 35 mM ethanol for 72 h or *SAR1A* siRNAs for 72 h and hepatocytes isolated from ethanol-fed rats. Lysates were normalized by β-actin (**a**) or giantin monomer (**b**). (**c**,**d**) Confocal immunofluorescence images of giantin (green) and PDIA3 (red) in VA-13 cells treated with 35 mM ethanol, *SAR1A* or *PDIA3* siRNAs for 72 h (**c**) and rat hepatocytes (**d**). The right panels show green, red and merged channels corresponding to the Golgi region (boxes). Nuclei were counterstained with DAPI (blue). All confocal images were acquired with same imaging parameters; bars, 10 μm. (**e**) Quantitation of the PDIA3-specific fluorescence signal colocalized with giantin in cells presented in (**c**) and (**d**). The average fluorescence intensity was measured as a mean ± SD of integrated fluorescence intensity (in arbitrary units, a.u.); n = 40 cells for each series of experiments. *p < 0.001. (**f**) PDIA3 Western blot of the lysates of VA-13 cells treated with 35 mM ethanol or *SAR1A* siRNA for 72 h and hepatocytes isolated from ethanol-fed rats; β-actin was a loading control. (**g**) Giantin Western blot of the lysates of VA-13 cells treated with *PDIA3* siRNAs for 72 h. Lysates were normalized by giantin monomer (top) or β-actin (bottom). (**h**) Western blot of Golgi fractions from VA-13 cells and hepatocytes. Golgi membranes were isolated from cells as described in Methods; 10 μg of Golgi membranes and 20 μg of postnuclear supernatant (PNS) from VA-13 cells, and 30 μg of Golgi membranes and 35 μg of PNS from rat hepatocytes were analyzed for PDIA3. The PNS samples were normalized by the PDIA3. The results shown are representative of three independent experiments.

Our recent observation of Golgi fragmentation in advanced prostate cancer cells^8^ prompted us to hypothesize that dysfunction of PDIA3 and subsequent impairment of giantin dimerization are responsible for ethanol-induced Golgi fragmentation. Our data indicate that the level of giantin and its dimer form was not only decreased in VA-13 cells after treatment with *SAR1A* siRNAs or ethanol but also in hepatocytes isolated from ethanol-fed rats (Fig. 4a,b). Additionally, ethanol treatment did not change the level of GM130 and GRASP65, supporting the reported finding that these Golgi proteins, independent of giantin, are involved in the incorporation of ER-to-Golgi carriers into the Golgi stacks^48^ (Supplementary Fig. S4a). These cells were then analyzed for PDIA3 by fluorescence microscopy. Quantification indicated that the fractions of PDIA3 that appeared in the Golgi area of both VA-13 cells and rat hepatocytes were identical in untreated and control siRNA-treated cells, but significantly higher than that from SAR1A-depleted cells or treated with ethanol (Fig. 4c–e). Further, in these cells, we detected a decrease of total PDIA3 by Western blotting (Fig. 4f). Predictably, depletion of PDIA3 in VA-13 cells induces Golgi fragmentation (Fig. 4c) and results in dedimerization of giantin (Fig. 4g)^8^. Importantly, the decrease of PDIA3 in the Golgi was confirmed in both ethanol treated VA-13 cells and rat hepatocytes by Western blotting (Fig. 4h), indicating that presence of PDIA3 in the Golgi requires SAR1A and COPII vesicles. Further, we confirmed that ethanol treatment results in alteration of COPII vesicles trafficking, as the presence of other components of COPII, including Sec23 and Sec24, in the Golgi were diminished (Supplementary Fig. S4b). However, it is important to note that at 48 h treatment with ethanol, when the level of SAR1A was already reduced (Supplementary Fig. S2c), the total amounts of both Sec23 and Sec24 were indistinguishable from that in untreated control (Supplementary Fig. S4c), showing that ethanol-induced alteration of COPII is initiated by downregulation of SAR1A. In light of these observations, we reasoned that PDIA3 was delivered to the Golgi by a COPII-dependent mechanism, and the decrease of PDIA3, total and Golgi-specific, might be caused by altered COPII function.

### The export of PDIA3 from the ER is regulated by COPII via SAR1A

**Figure 5:**
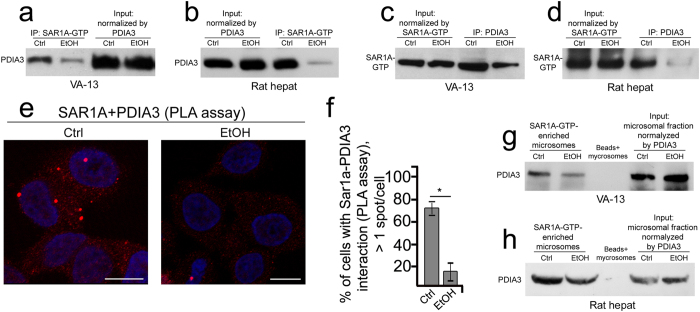
SAR1A and PDIA3 form complex in hepatocytes. (**a**,**b**) PDIA3 Western blot of complexes pulled down with anti-SAR1A-GTP Ab from the lysate of VA-13 cells treated with 35 mM ethanol for 72 h (**a**) or hepatocytes isolated from ethanol-fed rats (**b**). Lysates containing equal amounts of PDIA3 were used for Co-IP. (**c**,**d**) SAR1A-GTP Western blot of complexes pulled down with anti-PDIA3 Ab from the lysate of VA-13 cells treated with 35 mM ethanol for 72 h (**c**) or hepatocytes isolated from ethanol-fed rats (**d**). Lysates containing equal amounts of SAR1A-GTP were used for Co-IP. (**e**) Proximity ligation assay (PLA) of SAR1A and PDIA3 in VA-13 cells treated with 35 mM ethanol for 72 h, as analyzed by confocal microscopy; bars, 10 μm. (**f**) Quantification of PLA red spots (close proximity sites) scored per cell with more than one spots; n = 45 cells. The results are measured as a mean ± SD; *p < 0.001. (**g**,**h**) PDIA3 Western blot of microsomal fractions captures by Dynabeads M-270 Epoxy coated with SAR1A-GTP Ab. The microsomal fraction from ethanol-treated VA-13 cells (**g**) and hepatocytes isolated from ethanol-fed rats (**h**) were normalized by PDIA3. The uncoated beads exposed to microsomes served as a control.

To validate our hypothesis, we examined whether PDIA3 is incorporated into COPII vesicles and can be detected in a complex with SAR1A. First, co-immunoprecipitation assays were performed in both directions, PDIA3↔SAR1A-GTP, and produced the expected results: in both control VA-13 cells and rat hepatocytes SAR1A and PDIA3 formed complexes, but the fraction of active SAR1A associated with PDIA3 was reduced after ethanol treatment (Fig. 5a–d). Second, we analyzed the physical closeness of SAR1A and PDIA3 *in situ* by using a proximity ligation assay (PLA), which detects very close proximity between two proteins (below 30 nm)^49^. Red staining is considered positive for protein-protein interaction (Fig. 5e). We counted the cells with more than one red spot and found that in around 75% of control samples PDIA3 and SAR1A are present in a complex, while in cells treated with ethanol the positive signal was greatly reduced (Fig. 5e,f). Finally, to determine more precisely whether PDIA3 is a potential soluble cargo client for COPII, we performed immunoisolation of COPII vesicles from the rough ER-enriched microsomes using magnetic beads displaying an Ab to a SAR1A-GTP. As shown in Fig. 5g,h, PDIA3 was found in SAR1A-GTP-bearing microsomes in both VA-13 cells and rat hepatocytes, and much less PDIA3 was present in ethanol-treated samples, approximately 50% decrease after normalization to respective input.

Taken together, these data indicate that that PDIA3 leaves the ER in COPII vesicles, and in response to ethanol-induced alteration of COPII, PDIA3 is arrested in the ER and cannot assist in dimerization of giantin in the Golgi. Intriguingly, ethanol-induced Golgi fragmentation is accompanied by downregulation of not only SAR1A, but also PDIA3 and giantin. It is likely that these proteins are degraded through proteasome-mediated pathway, as ubiquitination of giantin, PDIA3 and SAR1A was detected at the earlier time of ethanol administration (Supplementary Fig. S5a–c), and inhibition of proteasome with MG-132 results in elevation of their level (Supplementary Fig. S5d). The marked reduction of SAR1A-GTP form in both VA-13 cells and rat hepatocytes (Fig. 2g,h) raised the suggestion that ethanol specifically suppresses activation of SAR1A rather than simply inhibiting its function. To check this idea, we examined whether overexpression of SAR1A or PDIA3 may rescue the condensed Golgi phenotype. As shown in Supplementary Fig. S6a and b, transfection with cDNA encoding SAR1A or PDIA3 in VA-13 cells does not alleviate the Golgi disorganization effect of ethanol, despite of elevated level of the respective proteins (Supplementary Fig. S6c and d).

### Ethanol-induced Golgi fragmentation results in accumulation of ASGP-R in the Golgi

**Figure 6:**
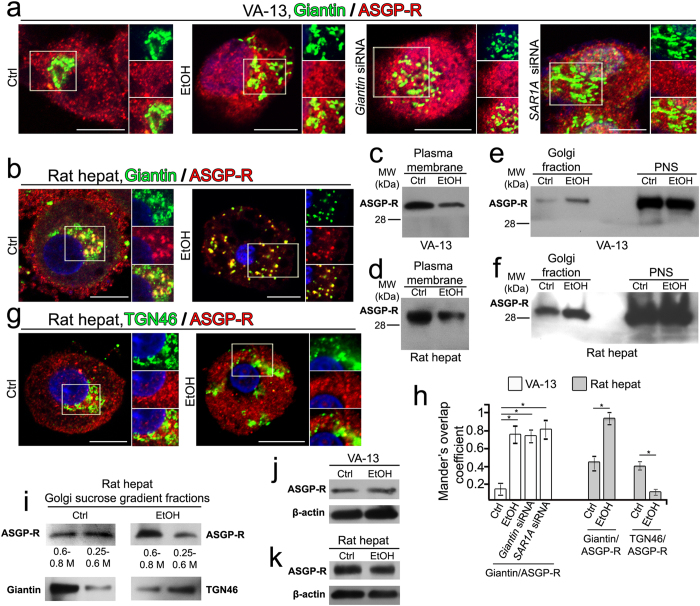
Ethanol treatment results in decrease of ASGP-R on the plasma membrane and its accumulation in the *cis-medial-*Golgi. (**a**) Immunostaining of giantin (green) and ASGP-R (red) in VA-13 cells after treatment with 35 mM ethanol for 72 h, and giantin (*GOLGB1*) and *SAR1A* siRNAs for 72 h. (**b**) Immunostaining of giantin (green) and ASGP-R (red) in hepatocytes isolated from ethanol-fed rats. (**c**,**d**) ASGP-R Western blot of the plasma membrane fraction of VA-13 cells treated with 35 mM ethanol for 72 h and hepatocytes isolated from ethanol-fed rats. Samples were normalized by the total protein amount. (**e**,**f**) Western blot of Golgi fractions from the VA-13 cells (**e**) and hepatocytes (**f**). Golgi membranes were isolated from cells as described in Methods; 25 μg of Golgi membranes and 45 μg of postnuclear supernatant (PNS) from VA-13 cells (**e**), and 35 μg of Golgi membranes and 50 μg of PNS from rat hepatocytes (**f**) were analyzed for ASGP-R. Both Golgi and PNS samples were normalized by the total protein amount. (**g**) Immunostaining of *trans*-Golgi marker, TGN46 (green) and ASGP-R (red) in hepatocytes isolated from ethanol-fed rats. All confocal images were acquired with same imaging parameters; bars, 10 μm. The right panels show green, red and merged channels corresponding to the Golgi region (boxes). (**h**) Quantification of Mander’s overlap coefficient reflecting the fraction of giantin or TGN46 which colocalizes with ASGP-R in cells presented in (**a**), (**b**) and (**g**); n = 45 cells for each series of experiments. The results shown are representative of three independent experiments and measured as a mean ± SD; *p < 0.001. Nuclei were counterstained with DAPI (blue). (**i**) ASGP-R Western blot of Golgi light (0.25 M/0.6 M) and heavy (0.6 M/0.8 M) fractions, corresponding to the *trans-* and *cis-medial*-Golgi, respectively, which were isolated by subfractionation of Golgi membranes from control and ethanol-fed rats on discontinuous (0.25/0.6/0.8 M) sucrose gradient. Giantin and TGN46 served as the *cis-medial-* and *trans-*Golgi markers, respectively. Samples were normalized by the total protein amount. (**j**,**k**) ASGP-R Western blot of the lysate of VA-13 cells treated with 35 mM ethanol for 72 h (**j**) and hepatocytes isolated from ethanol-fed rats (**k**); β-actin was a loading control.

We next investigated whether ASGP-R trafficking was affected when Golgi morphology was altered. To evaluate the level of intra-Golgi localization of ASGP-R, its colocalization with giantin (*cis-medial-*Golgi)^7^ was determined. In control VA-13 cells, about 17% colocalization of ASGP-R and giantin was observed (Fig. 6a,h). In contrast, ethanol treatment resulted in over 77% of giantin signal colocalizing with ASGP-R. Importantly, silencing either *GOLGB1* (giantin) or *SAR1A* genes mimics the effect of ethanol, i.e. the Mander’s coefficient of colocalization was 0.76 (76%) and 0.81 (81%), respectively (Fig. 6a,h), indicating that in cells lacking these proteins (either affected by ethanol or knockdown with siRNAs) ASGP-R is still detected in the Golgi. Interestingly, in control rat hepatocytes, the intra-Golgi fraction of ASGP-R (46% of colocalization with giantin) was much higher than that in VA-13 cells, while in hepatocytes isolated from ethanol-fed rats this parameter was increased to 93%, indicating that alcohol treatment leads to sequestration of the ASGP-R in the Golgi (Fig. 6b,h). The detection of more ASGP-R-specific punctae in the Golgi suggests that they arise as a consequence of the interrupted Golgi-to-plasma membrane trafficking. Indeed, Western blot analysis of the plasma membrane fractions isolated from VA-13 cells and rat hepatocytes showed that ethanol administration decreased the presence of ASGP-R at the plasma membranes (Fig. 6c,d). To validate these results, we measured ASGP-R in the Golgi membranes isolated from these cells. There was significantly more ASGP-R in the Golgi of ethanol-treated cells than that in untreated cells (Fig. 6e,f).

The next question we investigated is whether the sub-Golgi distribution of ASGP-R was altered. We analyzed the distribution of ASGP-R by comparing its colocalization with the *trans*-Golgi marker TGN46^50^, and the *cis-medial-*Golgi marker giantin^7^. In control cells, approximately 40% of TGN46 were detected with the ASGP-R, while ethanol administration significantly reduced this to 9% (Fig. 6g,h). To further determine the differences in the ASGP-R intra-Golgi distribution, we performed sucrose gradient (0.25 M/0.6 M/0.8 M) ultracentrifugation of Golgi membranes isolated from rat hepatocytes. Golgi light fraction (GLF), which contains the *trans-*Golgi, was collected at the 0.25 M/0.6 M sucrose interface, and the turbid band between 0.6 M and 0.8 M sucrose contained the Golgi heavy fraction (GHF, *cis-medial*-Golgi)^51^. As shown in Fig. 6i, while in control rat hepatocytes the ASGP-R was equally distributed between the Golgi stacks, in ethanol-treated samples the presence of ASGP-R in GLF was significantly less than in GHF. Importantly, under the same condition Golgi was depleted of PDIA3, implying that ASGP-R, contrary to PDIA3, is not sensitive to deficiency of COPII. Importantly, ethanol treatment did not change significantly the level of total ASGP-R in both VA-13 cells and hepatocytes (Fig. 6j,k), suggesting that ethanol does not effect on expression of ASGP-R but induces its intracellular redistribution. Thus, ethanol-specific Golgi fragmentation is dispensable for ER-to-Golgi trafficking of ASGP-R, but has a crucial effect on its intra-Golgi transportation, resulting in predominant deposition of ASGP-R in the *cis-medial-*Golgi, which, in turn, impairs its delivery to the plasma membrane.

### SAR1B is not involved in ethanol-induced Golgi disorganization

It is known that Sec13-Sec31 has a higher affinity for SAR1A than for SAR1B, implying the involvement of SAR1B paralog in the formation of an atypical large COPII vesicles^52^,^53^. However, we tested whether SAR1B depletion could cause Golgi disorganization in VA-13 cells. In control cells, the SAR1B Golgi-specific signal was weakly fluorescent, and knockdown of SAR1B had no apparent effect on Golgi organization (Supplementary Fig. S7a–c). Next, ethanol treatment did not affect expression of SAR1B, suggesting that dysfunction of SAR1B is not involved in alcohol-induced Golgi fragmentation (Supplementary Fig. S7d). In addition, we could not detect an interaction between SAR1B and PDIA3, as SAR1B IP does not coimmunoprecipitate PDIA3, and vice versa (Supplementary Fig. S7e and f). It is important to note that depletion of one paralog does not change the expression of other (Supplementary Fig. S7g and h). Finally, SAR1B knockdown has no effect on the presence of ASGP-R at the plasma membrane, as shown by Western blotting of plasma membrane fraction from VA-13 cells treated with *SAR1B* siRNAs (Supplementary Fig. S7i).

## Discussion

Alcoholic liver disease (ALD) is a major public health problem both in the USA and worldwide. ALD is the second leading cause of death among all the liver diseases and encompasses a spectrum of injury, ranging from alcoholic fatty liver to alcoholic cirrhosis^54^,^55^. Alcohol abuse is known to downregulate ASGP-R in the plasma membrane of hepatocytes, but the mechanism is not known. We present here the first evidence that ethanol treatment leads to downregulation of SAR1A which results in disorganization of the Golgi morphology and function. Using various approaches, we have shown that all of the observed ethanol-induced phenomena are found in SAR1A-depleted cells, including failed dimerization of giantin, arresting PDIA3 in the ER, large-scale alterations in Golgi architecture, and accumulation of ASGP-R in the Golgi.

We have found that treatment with ethanol leads to inactivation of SAR1A, as judged by the level of its active, GTP-bound form (Fig. 2g,h). Indeed, the overexpression of either SAR1A or PDIA3 failed to rescue the compact Golgi morphology, as a newly synthesized SAR1A molecules require an activation mechanism, and PDIA3 cannot serve for dimerization of giantin without an adequate amount of COPII. These results suggest that the SAR1A activation step is the one affected by ethanol treatment.

Importantly, SAR1-depletion effectively inhibits COPII vesicle formation^34^. In light of this, we postulate that ethanol administration alters function of COPII, as the levels of Sec23 and Sec24, two important core COPII proteins, were also decreased, although later than that of SAR1A (Supplementary Fig. S2c and Fig. S4c), confirming that Sec23-Sec24 heterodimer cannot be recruited to the ER membrane without active SAR1^56^. Meantime, we do not rule out the possibility that ethanol also may directly alter function of either inner (Sec23-Sec24) or outer (Sec13-Sec31) coat proteins.

How exactly does ethanol block activation of SAR1A? Perhaps, the key to understanding this phenomenon is the AMP-activated protein kinase (AMPK), the activity of which in the liver is reduced after ethanol administration^57^. On the other hand, the SAR1 recruitment to the membranes requires ATP^56^, and activation of SAR1 is suppressed by the protein kinase inhibitor H89^58^, indicating that formation of COPII vesicle is regulated by H89 sensitive kinases. At this point we can only speculate about SAR1A downregulation as a possible consequence of alcohol-induced blocking of kinases. Additionally, we do not exclude the possibility that the crucial effect of ethanol on COPII is also mediated through alteration of the Sec12, which catalyzes guanine exchange on SAR1, recruiting SAR1 to the ER membrane. Although there exists no direct evidence that Sec12 might be inactivated by ethanol, our data make this a likely scenario.

**Figure 7:**
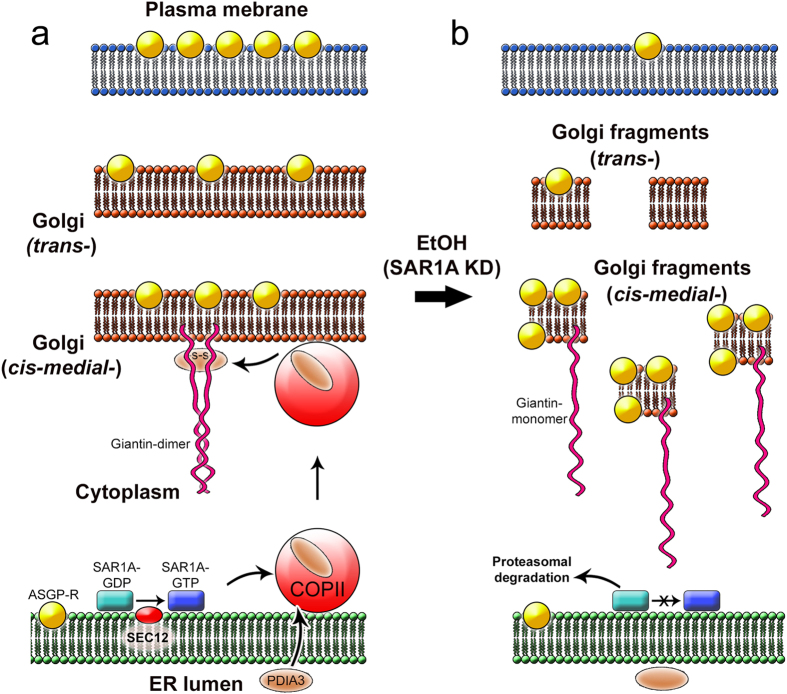
The proposed model of ethanol-induced SAR1A dysfunction and Golgi disorganization in hepatocytes. (**a**) Under normal conditions, Sec12 catalyzes guanine nucleotide exchange on SAR1A at the ER membrane, thus converting it to active GTP-bound form. This process initiates formation of COPII vesicles which is accompanied by capturing of PDIA3 (for simplicity, formation of the pre-budding complexes is not shown). After delivery to the Golgi membrane and fusion of COPII, PDIA3 catalyzes dimerization of giantin via its C-terminal^6^,^7^,^8^. (**b**) Ethanol administration, or SAR1A depletion, alters the exchange of GDP for GTP on SAR1A. Cessation of COPII formation blocks ER-to-Golgi trafficking of PDIA3, thus preventing giantin dimerization. Disruption of Golgi structure results in accumulation of ASGP-R in *cis-medial-*Golgi and proteasome-mediated degradation of SAR1A.

Previously, we have shown that giantin dimerization requires functional PDIA3, and knockdown of this enzyme results in Golgi fragmentation^8^. The classical view on function of PDI enzymes states that they are members of the protein disulphide isomerase family of oxidoreductases, which are involved in native disulphide bond formation in the ER^59^. However, this dogma was recently reformulated because different groups detected active PDIs outside of the ER, including Golgi^60^ and the cell surface^61^. Therefore, soluble ER proteins need the transporting carriers, and our data clearly demonstrate that PDIA3 employs COPII-dependent machinery to exit the ER. First, we were able to pull-down PDI3 by SAR1A Ab, and vice versa, indicating that these proteins are members of a complex in COPII. Second, the PLA confirmed their close proximity, and third, PDIA3 was detected in isolated SAR1A-specific microsomes. The precise mechanism of soluble ER cargo incorporation into COPII vesicles remains enigmatic. So far, the best explanation of this phenomenon is provided by Schekman’s group^62^. They demonstrated that both integral membrane and soluble ER cargo molecules form “specific complexes with activated Sar1 and Sec23/24 coat components before packaging into COPII vesicles”, which implies that “membrane-spanning adaptor proteins coordinate lumenal cargo packaging by cytosolic coat proteins”. Therefore, we anticipate the existence of a transmembrane adaptor that bridges PDIA3 into the vesicle during formation of COPII. In sum, our data allow us to infer that PDIA3, a soluble ER cargo, is transported to the Golgi membranes by COPII vesicles, where it assists in dimerization of giantin during Golgi biogenesis (Fig. 7a,b). Also, we believe that formation of functional giantin requires not only PDIA3, but also different chaperones and kinases^8^. Whether ethanol directly impacts their function and localization has yet to be investigated.

In this report, we have provided the explanation of why ethanol treatment reduces the fraction of ASGP-R at the plasma membrane of hepatocytes. Our results clearly show that ethanol metabolism disrupts Golgi-to-plasma membrane transportation of ASGP-R. Strikingly, we have found an increase of ASGP-R in *cis-medial*-Golgi, but only moderate presence in *trans*-Golgi (Fig. 7a,b). Importantly, ethanol treatment does not enhance the total level of ASGP-R, suggesting that the increase of its Golgi fraction is due to impaired Golgi-to-plasma membrane trafficking rather, than ER-to-Golgi transportation. Thus, in spite of SAR1A dysfunction, ASGP-R, unlike PDIA3, is successfully transported to the Golgi, demonstrating that *en route* to the Golgi ASGP-R utilizes a COPII-independent transport mechanism^34^. Also, while giantin serves as the docking sites for many Golgi targeting vesicles^4^,^5^, our data suggest that alteration in giantin structure is dispensable for Golgi targeting of ASGP-R. However, alternatively, ASGP-R may utilize the GM130-GRASP65 complex, which seems unaffected.

How does Golgi fragmentation impact ASGP-R trafficking to the *trans*-Golgi? Different possibilities can be envisioned here. It is known that intra-Golgi transportation of cargo proteins is mediated by COPI vesicles through tight cooperation with giantin^63^. Therefore, in the absence of functional giantin, the shuttling of COPI vesicles between fragmented Golgi cisternae might be altered. Also, we cannot rule out that alcohol may directly affect the formation of COPI vesicles.

It is important to note that ASGP-R, as with many endocytic receptors, is a transmembrane protein, and the phenomenon of ASGP-R deposition in the *cis-medial*-Golgi cannot necessarily be ascribed to the wide range of proteins secreted by cells. Their targeting to the plasma membrane and subsequent secretion may occur despite of Golgi disorganization. For instance, the carbohydrate deficient form of transferrin is still successfully secreted into the blood and serves as an excellent test for monitoring compliance of alcohol consumption^64^. Therefore, while “intra-Golgi highways” for plasma membrane receptors and secreted proteins appears to be segregated, the detailed mechanism needs to be clarified further.

We have recently shown that the cytoplasmic tails of Golgi resident glycosyltransferases are responsible for not only Golgi retention of these enzymes^65^,^66^ but also Golgi remodeling through interaction with the C-terminal cargo-binding region of the motor protein non-muscle Myosin IIA^67^. Golgi fragmentation in stressed cells^11^,^68^ and advanced cancer cells^8^ also occurs by this mechanism. In light of this, it is reasonable to assume that ethanol-induced Golgi fragmentation is also mediated by non-muscle Myosin IIA, and this possibility needs further investigation. Future analysis will also shed light on another important question whether ethanol-induced Golgi fragmentation is a process of random distribution of its membranes or programmed remodeling to create an alternative Golgi constitution.

Also, we might ask whether SAR1A downregulation is a general phenomenon of Golgi disassembly. Here, we observed that Golgi disruptive agent BFA does not follow this mechanism (Supplementary Fig. S3a–c). Our data indicate that dysfunction of SAR1A is the primary cause of the fragmentation of the Golgi, but we believe that every case should be independently investigated to determine whether alteration of COPII is the cause or the consequence of Golgi disassembly. We assume that deficiency of SAR1A results in impaired trafficking of other proteins involved in maintaining of Golgi morphology. However, giantin is the largest Golgi matrix protein and it controls intercisternal connection^6^,^7^,^8^, and the SAR1A-mediated pathway and subsequent formation of COPII directly regulates giantin dimerization through Golgi delivery of its enzyme PDIA3. Thus, given the similarity of ethanol- and SAR1A knockdown-induced Golgi disassembly, we conclude that SAR1A downregulation is the key event leading to ethanol-induced Golgi fragmentation.

Our results fit well with several other reports that have shown that blocking SAR1A activity results in Golgi disorganization^32^,^33^,^34^. Interestingly, the authors are unanimous in stating that the observed Golgi residential enzymes are sensitive to GTP- or GDP-locked mutations of SAR1A. However, the current story and our previous observations^5^,^8^ suggest that ER relocation of some glycosyltransferases is due to their altered Golgi docking mechanism rather than impaired COPII-dependent trafficking. On the other hand, the conclusions of these groups vary regarding the involvement of Golgi matrix proteins. Some authors believe that golgins are redistributed to the ER^2^,^33^, others support the idea that dysfunction of SAR1A does not alter their Golgi localization^32^. We propose, instead, the concept of selective responses, which exhibit divergent outcomes depending on the objects monitored. While alteration of COPII vesicles has no effect on some golgins, it may dramatically affect the function of others of which posttranslational modification requires the proteins be delivered to the Golgi by a COPII-dependent mechanism. Indeed, here we have found that ethanol-induced SAR1A downregulation impairs the dimerization of giantin and reduces its level (Fig. 4a,b), but does not change the level of two other important Golgi matrix proteins, GM130 and GRASP65 (Supplementary Fig. S4a). However, the detailed analysis of alcohol effect on the structure and localization of other golgins and GRASPs needs further analysis.

Here, we also show that treatment with ethanol leads to the reduced level of SAR1A. While the details of how COPII components are degraded still remain unknown, we assume that SAR1A may be degraded through proteasomal pathway, as ubiquitination of SAR1A was detected after only 24 h of ethanol exposure and inhibition of proteasomes results in accumulation of SAR1A. For now, this idea is supported by the publications which shown that other group of GTPases, Ras proteins, are subject to proteasomal degradation following the covalent conjugation of ubiquitin^69^,^70^. Here, we found that PDIA3 and giantin are also utilized by proteasomes, implying that in addition to downregulation of SAR1A, ethanol may promote oxidative stress, both by increased formation of reactive oxygen species (ROS) and by depletion of oxidative defenses in the cell^71^. Given that the ubiquitin proteasome system coordinates stress response^72^ and ER-stress reduces COPII vesicle formation^73^, it may provide an alternative explanation of these results.

Our data clearly show that SAR1A is the predominant paralog expressed in VA-13 cells. While lack of SAR1B can result in Golgi disassembly^34^, we could not detect significant changes in Golgi morphology or reduced level of plasma membrane-associated ASGP-R in VA-13 cells lacking SAR1B after *SAR1B* siRNA treatment (Supplementary Fig. S7a and i). Further, the level of SAR1B seems stable after ethanol treatment, and SAR1B does not form complexes with PDIA3 (Supplementary Fig. S5d–f). Taken together, these data lead us to suggest that the expression and function of SAR1 paralogs may be tissue-specific^53^.

In sum, our findings demonstrate that the normal Golgi organization is likely to be an overriding condition for successful and complete targeting of ASGP-R to the plasma membrane. Given that ASGP-R-deficient hepatocytes are more susceptible to ethanol-induced apoptosis^74^, and the severity of ALD is correlated with the number of apoptotic cells^75^, the ethanol-induced Golgi fragmentation may be a potential contributor to the death of hepatocytes and initiation of ALD.

## Methods

### Antibodies and reagents

The primary antibodies were: a) rabbit polyclonal – giantin, PDIA3, ASGP-R1 (Abcam); b) rabbit monoclonal – GM130, SAR1B (Abcam); c) mouse monoclonal – SAR1A, giantin and TGN46 (Abcam), β-actin (Sigma), GRASP65, Sec24d (Santa Cruz Biotechnology), giantin (Abcam); goat polyclonal – COPII (Sec23a, Abcam). The secondary antibodies (Jackson ImmunoResearch) were: a) HRP-conjugated donkey anti-rabbit, donkey anti-mouse, and donkey anti-goat for Western-blotting; b) donkey anti-mouse Alexa Fluor 488 and anti-rabbit Alexa Fluor 594. Pyrazole (Sigma), MG-132 (EMD Chemicals) and Brefeldin A (Sigma) were dissolved in dimethyl sulfoxide (DMSO) immediately before use. Cells treated with a corresponding concentration of DMSO served as controls. The regular working concentrations were: pyrazole – 5 mM for 72 h, MG-132 – 2.5 μM for 24 h and Brefeldin A – 36 μM for 1 h.

### Cell culture, ethanol and acetaldehyde administration

HepG2 cells, obtained from ATCC (Rockville, MD), were grown in Dulbecco’s modified Eagle medium (DMEM) as described earlier^41^. HepG2 cells transfected with mouse alcohol dehydrogenase1 (ADH1) (VA-13 cells) and mouse ADH1 and human cytochrome P450 2E1 (CYP2E1) (VL-17A cells) were established and maintained as previously described^41^,^42^. Twenty-four hours after seeding cells (at ∼75% confluence), culture media were changed too media containing 35 mM ethanol for another 72 h. The media was replaced every 12 h to mitigate ethanol evaporation. Control cells were seeded at the same time as treated cells, and maintained under the same conditions but without the addition of ethanol. In other experiments, cells were treated with the ADH inhibitor pyrazole (5 mM) plus ethanol for 72 h. Cells treated with only pyrazole served as a control. The 100 μM stock solution of acetaldehyde (Sigma) was prepared daily in PBS. The cells were then incubated with acetaldehyde for up to 3 days, and the medium was replaced every 12 h to prevent evaporation of acetaldehyde. The control cells were treated with corresponding amount of PBS.

Primary rat hepatocytes from control and ethanol-fed animals were prepared from male Wistar rats. Rats weighing 140–160 g were purchased from Charles River Laboratories. Initially, the animals were fed a Purina chow diet and allowed to acclimate to their surroundings for a period of 3 days. Then the rats were paired according to weight and fed either control or ethanol containing (36% of calories) Lieber-DeCarli diet for periods of 5–7 weeks (Dyets, Inc). This project was approved by the Institutional Animal Care and Use Committee of the Omaha Department of Veterans Affairs Medical Center and the University of Nebraska Medical Center. The animals were handled in accordance with applicable local and federal regulations concerning laboratory animals. Hepatocytes were obtained from the livers of control and ethanol-fed rats by a modified collagenase perfusion technique previously described by Casey *et al.* (1987)^30^. The isolated cells were washed with Krebs-Ringer buffer, purified over a 35% Percoll gradient, and equilibrated at 37 °C for 45 min in William’s buffer containing 0.1% BSA. Then the hepatocytes were washed, counted, resuspended in William’s/BSA medium, and placed on ice until they were divided for the various experiments.

### Immunoprecipitation (IP) and siRNA transfection

The IP steps were performed using Pierce Co-Immunoprecipitation Kit (Thermo Scientific) according to manufacturer’s instructions. Mouse and rabbit non-specific IgG (Jackson ImmunoResearch) were used as non-specific controls. All cell lysate samples for IP experiments were normalized by target proteins. To determine whether the target protein is loaded evenly, the input samples were preliminary run on a separate gel with different dilution of control samples vs. treated and then probed with anti-target protein Abs. The intensity of obtained bands was analyzed by ImageJ software, and samples with identical intensity were subjected for IP. The active, GTP bound form of SAR1A was detected by Sar1 Activation Assay Kit (Abcam). Briefly, the anti-active SAR1A mouse monoclonal Ab was incubated with cell lysates, and then the bound active SAR1A was pulled down by protein A/G agarose and the IP complexes were analyzed by Western blotting using anti-SAR1A rabbit polyclonal Ab. The pool of 3 different siRNA duplexes targeting the mRNA sequences corresponding to the product of each SAR1 paralog (*SAR1A, SAR1B*), *Sec24d, GOLGB1* (giantin), and scrambled on-targetplus smartpool siRNAs were purchased from Santa Cruz Biotechnology. The sequences of the sense strands were: for *SAR1A* (a) 5′GGA AGU GCA UGC AUU UCG U dTdT (b) 5′GCA UAU AUG UCC UAC UUC A dTdT (c) 5′GGA AUG ACC UUU ACA ACU U dTdT; for *SAR1B* (a) 5′GAC AAC AUG UCC CAA CAU U dTdT (b) 5′GAG AUU UGA UUG CUC AAC A dTdT (c) 5′CAA CUU UCC AGC AGU ACA U dTdT; for the control - 5′UUC UCC GAA CGU GUC ACG U dTdT. Cells were transfected with 100–200 nM siRNAs using Lipofectamine RNAi MAX reagent (Life Science technologies). Full length human *PDIA3* cDNA in pGEc2-DEST vector and full length human *SAR1A* in pENTR223 vector were obtained from DNASU Plasmid Repository. Transient transfection of VA-13 cells was carried out using the Lipofectamine 2000 (Life Science technologies) following the manufacturer’s protocol.

### Immunoisolation of SAR1A-enriched vesicles

Isolation of microsomes was performed using Endoplasmic Reticulum Isolation Kit (Sigma) according to manufacture’s protocol. Briefly, cells were suspended in Hypotonic Extraction Buffer and incubated for 20 minutes at 4 °C to allow the cells to swell. Then, after centrifugation at 600xg for 5 minutes and homogenization, homogenate were centrifuged at 1,000xg for 10 minutes (4 °C). The obtained postnuclear supernatant were centrifuged at 12,000xg for 15 minutes (4 °C), and supernatant (post mitochondrial fraction) was further subjected to isolation of rough endoplasmic reticulum (RER) using precipitation by 8 mM CaCl_2_.

The coupling of SAR1A-GTP-Ab (Sar1 Activation Assay Kit, Abcam) to Dynabeads M-270 Epoxy was performed using Dynabeads Antibody coupling kit (Life Science Technologies) according to the manufacture’s instruction. The RER-enriched microsomes (100 μg protein) were added per 2 × 10^7^ of coated beads in Dynal buffer A (PBS, pH 7.4, 2 mM EDTA, 5% BSA) and incubated overnight at 4 °C with rotation^76^. After thorough washing in PBS, beads were isolated by the Magnetic Separation Rack (New England BioLabs) and the samples were solubilized in SDS sample buffer for subsequent analysis by Western blot. Negative controls were prepared by exposing uncoated 2 × 10^7^ Dynabeads M-270 Epoxy to 100 μg protein of the RER-enriched microsomes.

### Analysis of immunofluorescence stained cells by confocal microscopy

Cells were immunofluorescence-labeled as described previously^68^. For some figures, image analysis was performed using Adobe Photoshop and the ImageJ. Statistical analysis of colocalization was performed by ImageJ calculating the Mander’s overlap coefficient, corresponding to the fraction of green pixels that overlap with red pixels in relation to the total green pixels^77^.

### *In situ* Proximity Ligation Assay

The assay was performed using the Duolink kit (Olink Bioscience) according to the manufacturer’s protocol. Briefly, VA-13 cells were treated with mouse anti-SAR1A and rabbit anti-PDIA3 antibodies, and then with oligonucleotide-conjugated anti-mouse minus and anti-rabbit plus proximity ligation assay secondary probes. The oligonucleotides were used to generate circular DNA, which was then amplified and tagged with a red fluorescence dye. Following DAPI staining of the nuclei, the cells were examined by confocal fluorescence microscopy. The red spots, which represent interaction, from 20 randomly selected images were counted and plotted.

### Isolation of Golgi membrane fractions by sucrose gradient centrifugation

Golgi membrane fractions were isolated using methods described previously^68^. For preparation of the Golgi heavy and light fractions, the isolated Golgi membranes were subject to centrifugation on 0.25/0.6/0.8 M Tris-HCl (pH 7.4) sucrose gradients^51^. The Golgi membranes were adjusted to 2.2 ml of 0.8 M sucrose and loaded at the bottom of tube, which was followed by 1.1 ml of 0.6 M sucrose and then 550 μl of 0.25 M sucrose. The gradients were centrifuged for 4 h at 42,000 rpm (4 °C) in a SW60 rotor (Beckman Coulter). The turbid band between 0.6 M and 0.8 M sucrose, which contains the Golgi heavy fraction corresponding to *cis-medial*-Golgi, and the 0.25 M/0.6 M sucrose interface, which contains Golgi light fraction corresponding to the *trans*-Golgi, were collected. The giantin and TGN46 served as a markers for *cis-medial*-7 and *trans*-Golgi^50^, respectively.

### Transmission Electron Microscopy

EM was performed using protocols described previously^8^.

### Plasma membrane protein isolation

Plasma membranes were isolated using Pierce Chemical kit (Thermo Scientific) according to their protocol.

## Additional Information

**How to cite this article**: Petrosyan, A. *et al.* Downregulation of the small GTPase SAR1A: a key event underlying alcohol-induced Golgi fragmentation in hepatocytes. *Sci. Rep.* **5**, 17127; doi: 10.1038/srep17127 (2015).

## Supplementary Material

Supplementary Information
