# Supplementary material for: Downregulation of the small GTPase SAR1A: a key event underlying alcohol-induced Golgi fragmentation in hepatocytes
Source: Sci Rep. 2015 Nov 26;5:17127. doi: 10.1038/srep17127 (PMC4660820; doi:10.1038/srep17127)

Downregulation of the small GTPase SAR1A: a key event underlying alcohol-induced Golgi fragmentation in hepatocytes

Armen Petrosyan[1*], Pi-Wan Cheng[1,3] , Dahn L. Clemens[2,3] & Carol A. Casey[2,3]
[1]Department of Biochemistry and Molecular Biology, College of Medicine, and [2]Department of Internal Medicine, University of Nebraska Medical Center, Omaha, NE, USA, [3]Omaha Western Iowa Health Care System, VA Service, Department of Research Service, Omaha, NE, USA.

Supplementary materials

Figure S1. Immunostaining of GRASP65 (green) and GM130 (red) in VA-13 cells treated with 35 mM ethanol for 72 h. The right panels show high magnifications of merged channel corresponding to the Golgi region (boxes). Nuclei were counterstained with DAPI (blue); bars, 10 μm.

Figure S2. (a) Immunostaining of giantin in cells treated with 35 mM ethanol for 24 and 48 hours. Nuclei were counterstained with DAPI (blue); bars, 10 μm. (b) Quantification of cells with fragmented Golgi in cells presented in (a). Results are expressed as mean ± SD; n = 35 cells for each series of experiments; *, $p<0.001$. (c) SAR1A Western blot of the lysates of VA-13 cells treated with ethanol for 24 and 48 hours; β-actin was a loading control.

Figure S3. (a) Immunostaining of giantin in VA-13 cells treated with 36 μM Brefeldin A (BFA) for 1 h. Nuclei were counterstained with DAPI (blue); bars, 10 μm. (b) Quantification of cells with fragmented Golgi in cells presented in (a). Results are expressed as mean ± SD; n = 35 cells. (c) SAR1A Western blot of the lysates of VA-13 cells treated with 36 μM Brefeldin A (BFA) for 1h; β-actin was a loading control.

Figure S4. (a) GM130 and GRASP65 Western blot of the lysates of VA-13 cells treated with 35 mM ethanol for 72 h; β-actin was a loading control. (b) Sec23a and Sec24d Western blot of the Golgi fraction (normalized by GM130) from VA-13 cells treated with 35 mM ethanol for 72 h. (c) Sec23a and Sec24d Western blot of the lysates of VA-13 cells treated with 35 mM ethanol for 48 h; β-actin was a loading control.

Figure S5. (a-c) Ubiquitin western blot of complexes pulled down with anti-SAR1A (a), anti-giantin (b) and anti-PDIA3 (c) Abs from the lysate of VA-13 cells treated with 35 mM ethanol for 24 h and 48 h, respectively. Lysates containing equal amounts of SAR1A (a), giantin (b) and PDIA3 (c) were used for IP. (d) Western blot of giantin, PDIA3 and SAR1A of the lysates of VA-13 cells treated with DMSO or 2.5 μM MG-132 for 24 h.

Figure S6. Overexpression of either SAR1A or PDIA3 in ethanol-treated cells does not prevent Golgi fragmentation. (a) Immunostaining of giantin in EtOH-treated VA-13 cells transfected with SAR1A or PDIA3 cDNAs. Cells were transfected with SAR1A or PDIA3 cDNA, and after 12 h were treated with 35 mM ethanol for another 72 h. (b) Quantification of cells with fragmented Golgi in cells presented in (a). (c, d) SAR1A (c) and PDIA3 (d) Western blot of the

lysates of VA-13 cells transfected with a full length SAR1A or PDIA3 cDNA, respectively; β-actin was a loading control.

Figure S7. SAR1B is dispensable for ethanol treatment-induced Golgi fragmentation. (a) SAR1B Western blot of the lysates of VA-13 cells treated with control and *SAR1B* siRNAs for 72 h; β-actin was a loading control. (b) Immunostaining of SAR1B (green), and giantin (red) in VA-13 cells treated with *SAR1B* siRNAs. Nuclei were counterstained with DAPI (blue). All confocal images were acquired with same imaging parameters; bars, 10 μm; (c) Quantification of cells with fragmented Golgi in cells presented in (b). Results are expressed as mean ± SD. (d) SAR1B Western blot of the lysates of VA-13 cells treated with 35 mM ethanol for 72 h; β-actin was a loading control. (e) PDIA3 western blot of complexes pulled down with anti-SAR1B Ab from the lysate of VA-13 cells. (f) SAR1B western blot of complexes pulled down with anti-PDIA3 Ab from the lysate of VA-13 cells. (g, h) SAR1A (g) and SAR1B (h) Western blot of the lysates of VA-13 cells treated with control, *SAR1A* and *SAR1B* siRNAs; β-actin was a loading control. (i) ASGP-R Western blot of the plasma membrane fraction of VA-13 cells treated with control or *SAR1B* siRNAs. Samples were normalized by the total protein amount.

Supplemental figure 1.

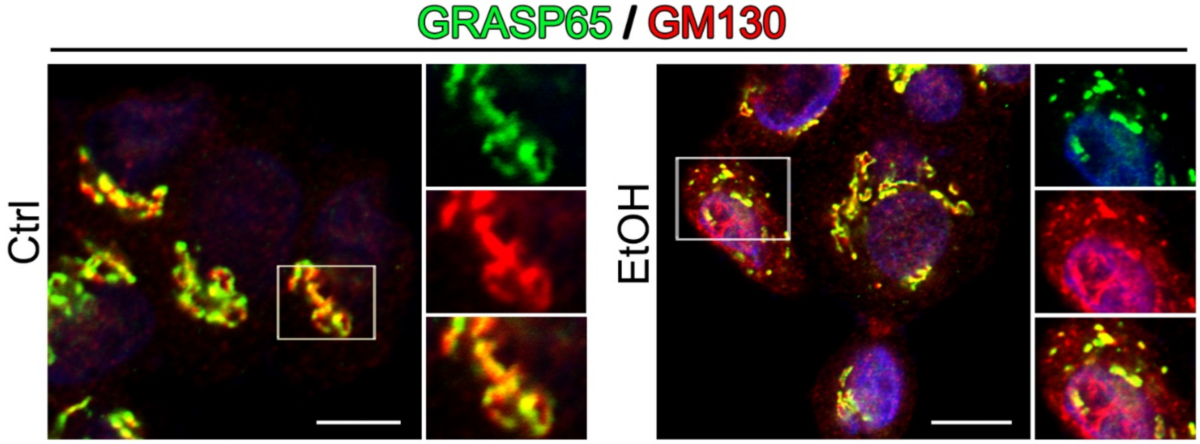

Supplemental figure 2.

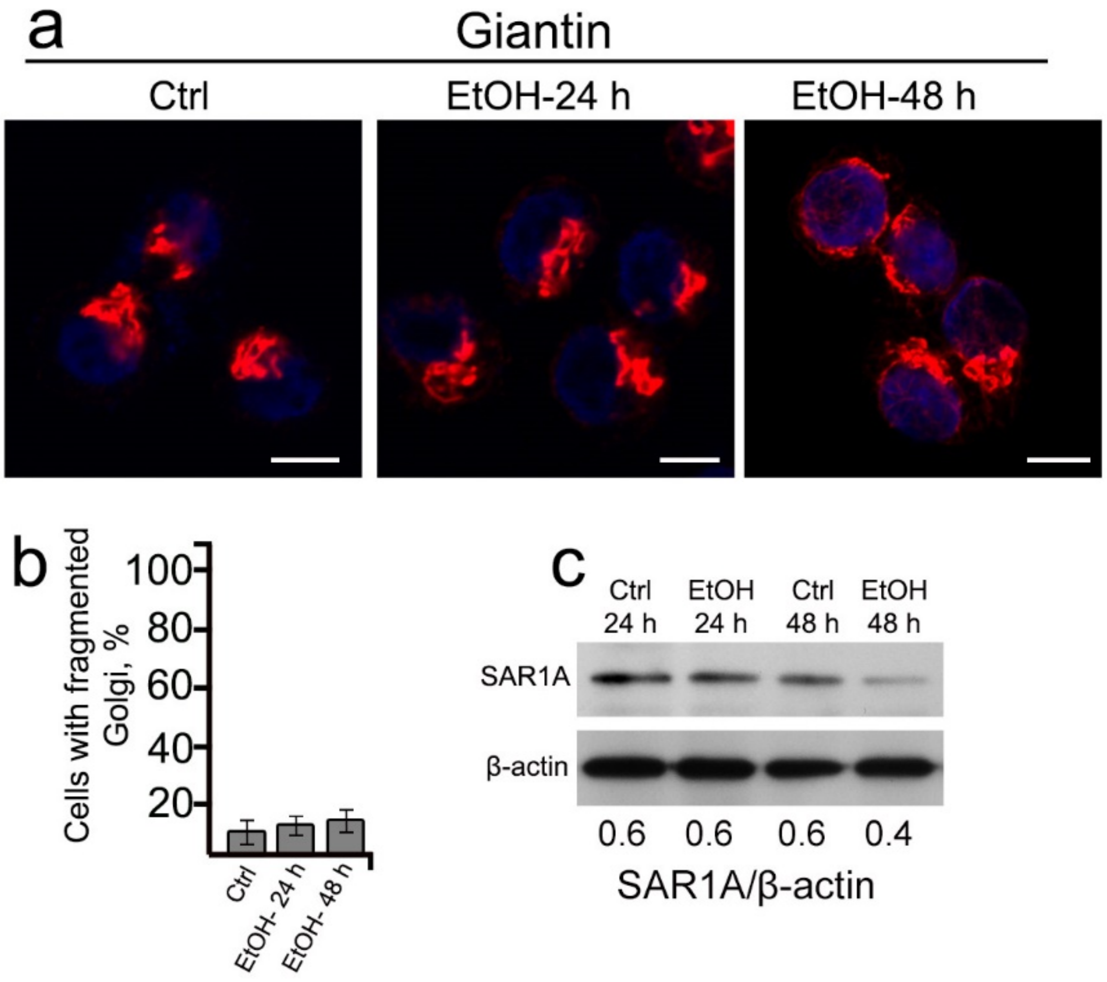

Supplemental figure 3.

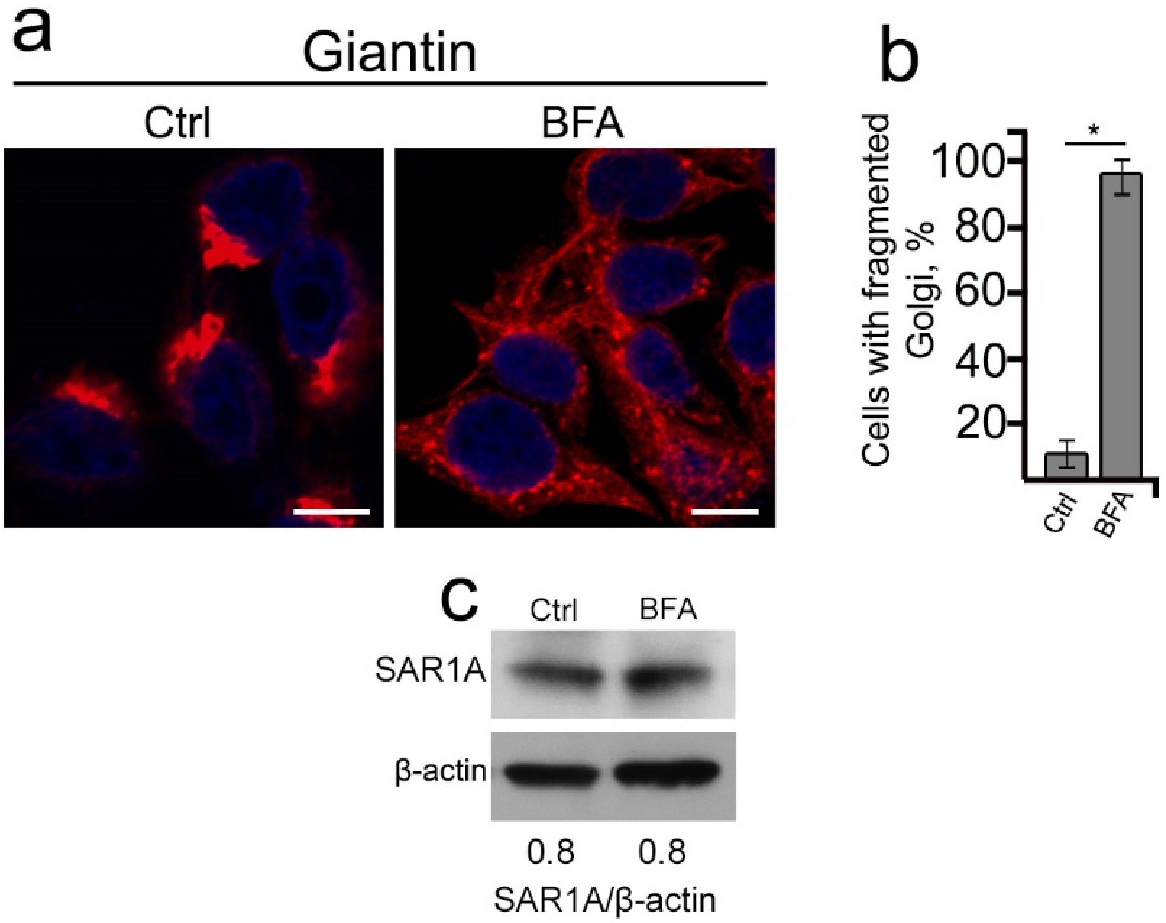

Supplemental figure 4.

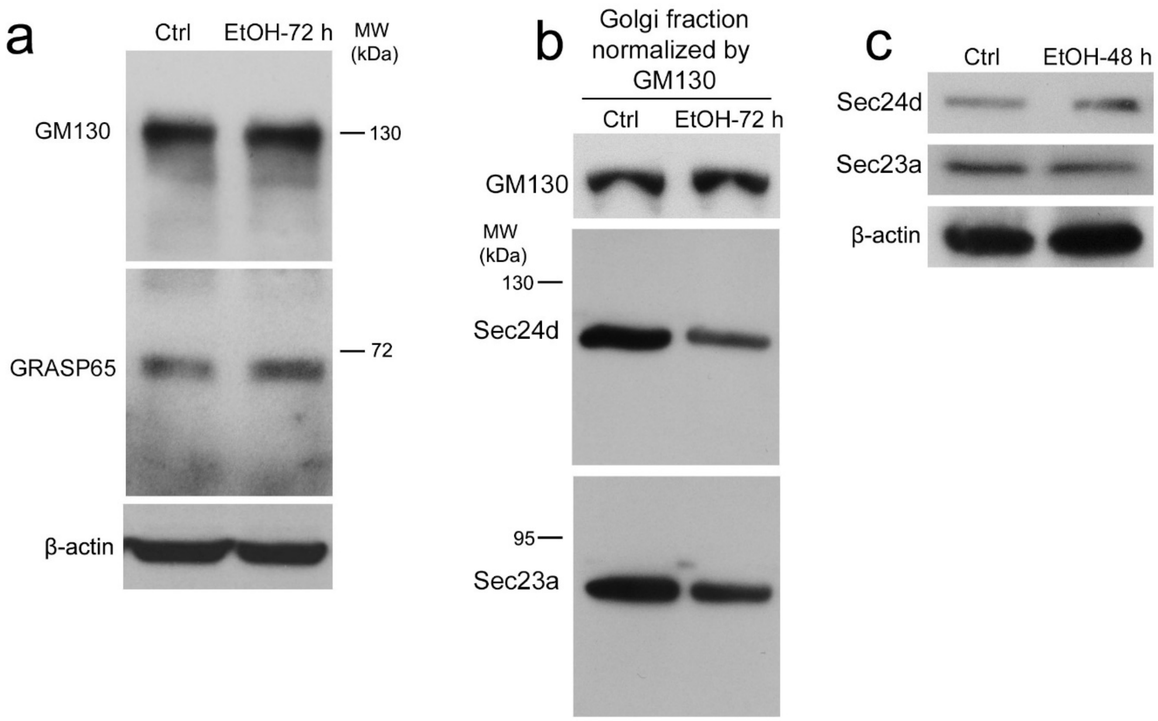

Supplemental figure 5.

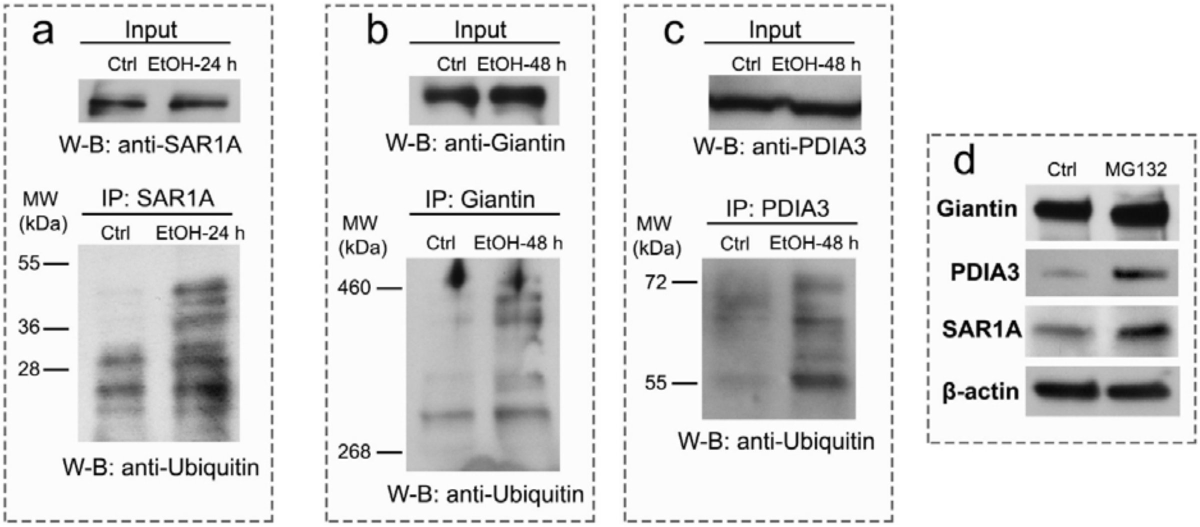

Supplemental figure 6.

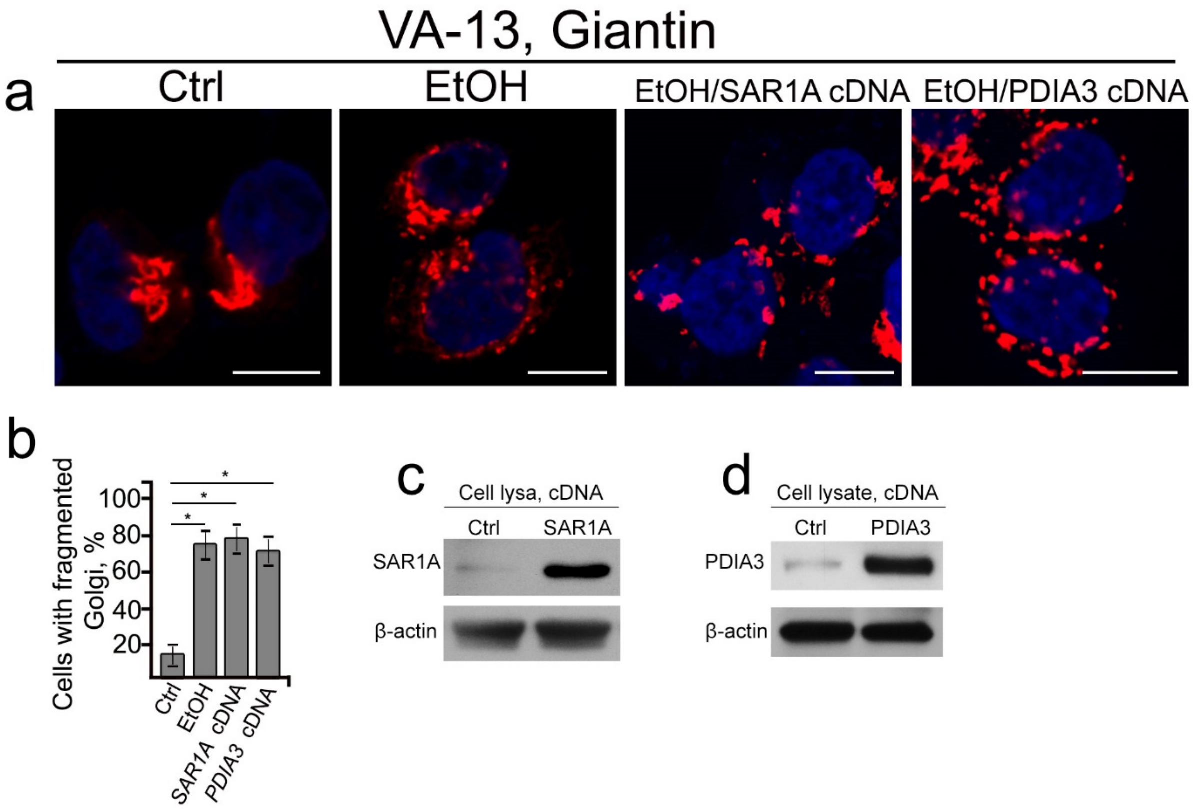

Supplemental figure 7.

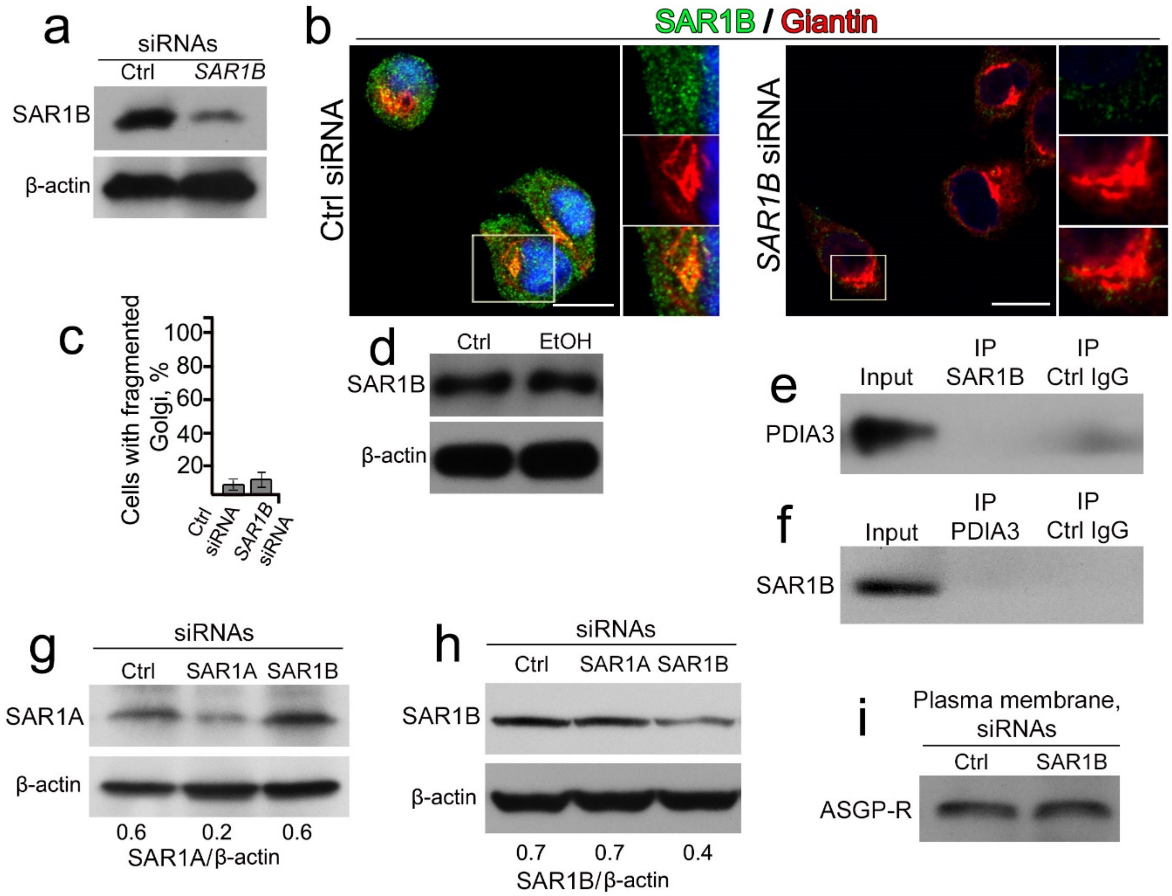

Supplement: Supplementary Information [file srep17127-s1.pdf]
